# Two-Generation Epsilon-Sarcoglycan Gene (SGCE) Mutation-Associated Myoclonus-Dystonia (DYT-SGCE) Misdiagnosed as Tourette’s Syndrome: A Case Series

**DOI:** 10.7759/cureus.45289

**Published:** 2023-09-15

**Authors:** Laura Surillo-Dahdah, Carlos A Morfi-Pagán

**Affiliations:** 1 Department of Neurology, Institute of Neuroscience, Manatí Medical Center, Manatí, PRI; 2 Department of Medical Education, Ponce Health Sciences University, Ponce, PRI

**Keywords:** myoclonus-dystonia syndrome, sgce, dyt-sgce, myoclonus, focal dystonia

## Abstract

This case series provides a diagnosis of myoclonus-dystonia syndrome (MDS) in two patients whose original presentation was thought to be Tourette’s syndrome. The first patient presented with dystonia and myoclonus, which progressively worsened with age, and was diagnosed with an epsilon-sarcoglycan gene (SGCE) mutation. The patient’s father, who was diagnosed in his childhood with Tourette’s syndrome, also received genetic testing, which proved that to be a misdiagnosis and confirmed that he was the carrier of the SGCE mutation. Both patients were subjected to a levodopa trial, which proved to be an effective treatment. To our knowledge, these are the first reported cases of heterozygous pathogenic mutation of SGCE in Puerto Rico.

## Introduction

Myoclonus-dystonia syndrome (MDS) due to epsilon-sarcoglycan gene (SGCE) mutations (DYT-SGCE) is a rare movement disorder that affects the encoding of ε-sarcoglycan, a membrane protein widely expressed in the cerebello-thalamic pathway. DYT-SGCE mutations follow an autosomal dominant inheritance pattern with penetrance based on the parental origin [1]. MDS has a clinical presentation before the second decade of life with equal predisposition to both sexes [2]. It generally presents with myoclonus of the upper limbs and neck with cervical dystonia and writer’s cramp [1]. DYT-SGCE mutations have also been linked to psychiatric disorders such as anxiety and obsessive-compulsive disorder [3]. Currently, there are no prevalence studies of MDS due to the rarity of cases. To our knowledge, this case series presents two patients who are the first cases of MDS due to a SGCE mutation formally presented in Puerto Rico.

## Case presentation

A 13-year-old female patient was brought to our movement disorders clinic due to progressive worsening jerking movement of arms and legs with right torticollis with right shoulder elevation. She also had focal segmental myoclonic jerks of her arms and trunk with occasional twisting and posturing. Cranial nerve testing was normal, and motor and sensory testing had no abnormalities. Dystonic posturing was also observed as finger spooning. The patient’s past medical history had significant acute events of akinesia without any pertinent symptoms. Myoclonus was first observed with intentional movement at the age of four. Her pediatrician’s original impression was that she was mimicking her father, aged 51 years, who was diagnosed during childhood with Tourette’s syndrome due to similar left torticollis, left arm myoclonus, eye blinking, and interrupted speech. At the age of seven years, the patient was referred to a pediatric neurologist for further workup due to worsening symptoms. At that moment, an electroencephalogram (EEG) found abnormal generalized polyspikes. Symptoms continued to worsen, and at 10 years old, the involuntary movement involved her whole body without loss of consciousness. At age 13, she was referred to our clinic. The frequency of myoclonic movements increased when she was anxious or in social situations. Her family history was unremarkable besides her father’s Tourette’s syndrome diagnosis.

**Figure 1:**
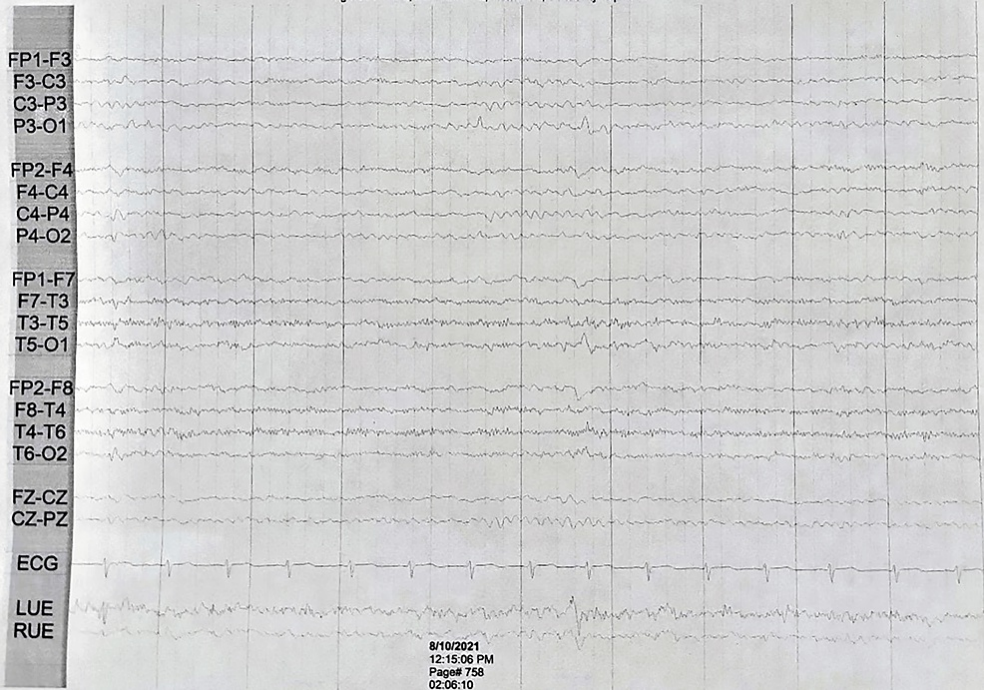
The 13-year-old female patient’s 24-hour ambulatory electroencephalogram Electrodes were arranged according to the international 10-20 configuration. The patient had normal sleep architecture. There are no epileptiform discharges or asymmetries observed during the study.

Wilson’s disease workup, serum copper (103 µg/dL), and ceruloplasmin (26 mg/dL) were within normal limits. Vitamin B-12 levels (456 pg/dL) were also found within normal limits. After the patient interview, she was ordered a digital 24-hour ambulatory EEG, which presented no epileptiform discharges or asymmetries during the study (Figure 1). Brain magnetic resonance imaging (MRI) without contrast presented no acute intracranial process nor cortical abnormalities. The patient confirmed in the interview that the jerk-like movements were not urges nor provided any relief after completing them. No vocal tics were reported. The Diagnostic and Statistical Manual of Mental Disorders, Fifth Edition, requires there to be vocal tics for the diagnosis of Tourette’s syndrome. The lack of those makes the diagnostic inaccurate.

The patient had been previously treated for myoclonus with levetiracetam 1,000 mg daily, valproic acid 15 mg/kg/day, and clonazepam 1 mg/kg/day PO. The suspected tics had been treated with topiramate 400 mg extended-release (ER) daily, pimozide 2 mg/day, Risperdal 3 mg/day PO, and guanfacine 4 mg/day BID-QID without any marked improvement in symptoms. At the clinic, both the patient and her father were treated with carbidopa/levodopa 10 mg/100 mg PO TID, which lowered the frequency of myoclonus and decreased the dystonia. The next dosage attempted was 25 mg/100 mg PO TID, which presented increased effectiveness with no dyskinesia or secondary effects. The last dose attempted was 25 mg/250 mg PO TID, which caused muscle stiffness and discomfort. Both patients continued treatment with 25 mg/100 mg PO TID carbidopa/levodopa since it presented the most efficacy.

Whole exome sequencing (WES) detected an SGCE heterozygous pathogenic mutation (c.662+2T>A) along with a FOXRED1 heterozygous pathogenic mutation (c.612_615dup (p.Ala206serfs*15)). Positive genetic results for SGCE mutation provided the diagnosis of an autosomal dominant dystonia (DYT11) or myoclonus-dystonia.

Genetic assessment of the patient and her father’s past diagnosis of Tourette’s syndrome indicated a high probability of the father being the carrier of the SGCE pathogenic mutation. The 51-year-old male had presented involuntary movements that worsened in adolescence and remain present. Vocal tics were not reported during childhood or adolescence; he disclosed having a history of occasional eye blinking with recurrent speech interruption. On physical evaluation, he presented left torticollis and worsening jerky dystonia when looking right with relief when looking left. He had left shoulder arm myoclonic jerks. He denied an urge or feelings of relief after completing the movement.

**Figure 2:**
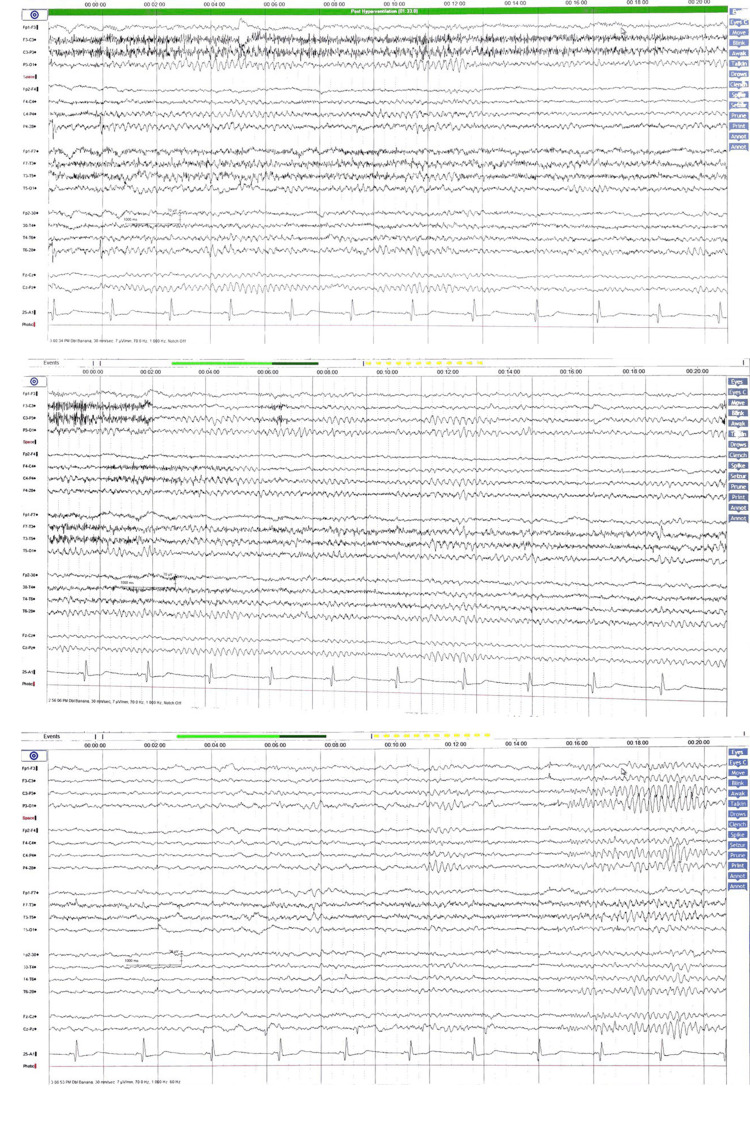
The 51-year-old male patient’s 24-hour ambulatory electroencephalogram Electrodes were arranged according to the international 10-20 configuration. The patient had normal sleep architecture. There are no epileptiform discharges or asymmetries observed during the study.

Brain MRI presented no abnormalities. Past EEGs did not show epileptogenic focus (Figure 1 and Figure 2). Levodopa trials were done, in which the patient’s dystonia was responsive to L-dopa. Just like his daughter, due to scarcity of genetic services and complex symptoms, a whole exon sequencing was completed, which confirmed that he was the carrier of the SGCE mutation, which was the same heterozygous pathogenic mutation.

Since both patients were responsive to levodopa trials, carbidopa/levodopa 25 mg/100 mg two tablets PO TID was continued as the main treatment for dystonia and myoclonus in both cases with follow-up visits every three months. Both patients were also treated with electromyographic-guided onabotulinum toxin injections for their cervical dystonia. For the daughter, injections of 25 units were applied to the left sternocleidomastoid muscle and 15 units to the right levator scapulae. Meanwhile, the father was injected with 25 units to the left sternocleidomastoid and 20 units to the right levator scapulae. Doses were to be adjusted and optimized as needed in future injections every three months.

## Discussion

In this case report, we present two patients with a delayed diagnosis of myoclonus-dystonia syndrome, one of whom was originally thought to be Tourette’s syndrome. MDS is a rare, autosomal dominant disease. Symptoms are commonly observed in the first two decades of life [1]. MDS is caused by a heterozygous loss-of-function mutation in the gene that encodes epsilon-sarcoglycan (SGCE) on chromosome 7q21.3 [4]. Previous cases have reported variations in mutation, yet phenotype-genotype correlation analysis did not show significant differences in the severity of dystonia and myoclonus in patients with insertion, deletion, truncation, or splicing mutations [5]. Genetic studies have also reported that the inheritance pattern within MDS families is strongly correlated to maternal impression, yet there are few cases in which irregular imprinting and mosaicism have led to varied phenotypic presentation according to the tissues that were taken for testing [4].

An MDS diagnosis is highly dependent on the use of genetic testing due to the heterogeneity of onset, kinetic manifestation, and psychiatric disorders. It is not uncommon for rare diseases such as MDS to have a delayed diagnosis. It is also highly likely for psychiatric comorbidities to go undiagnosed [6]; an incomplete clinical presentation can make difficult cases even harder to diagnose. Nearly 95% of cases of SGCE mutations have an age of onset of 10 years or younger with no cases reported over the age of 20 [7]. This proposes that the age of onset is a strong predictor of MDS due to an SGCE mutation [2]. Previous case reports of SGCE myoclonus-dystonia had a delayed diagnosis decades later due to symptoms being confused with Tourette’s syndrome [8]. Due to the complex clinical presentation, a novel eight-step diagnostic algorithm for myoclonus has been proposed, which can lead to a proper diagnosis of MDS [9]. To our knowledge, these are the first cases of SGCE myoclonus-dystonia recorded in Puerto Rico.

Previous studies presented that up to 5% of patients with childhood-onset generalized dystonia were dopa-responsive dystonias (DRD) [3]. Both patients presented positive outcomes from levodopa trials and have continued pharmacological treatment for dystonia and myoclonus. Botulinum toxin has been in use since the 1980s as a treatment for dystonic muscle weakening and improving muscle tone [3]. In our case, both patients have also received one session of Botox injections on sites that presented dystonia to alleviate tension. The patients will continue the same line of treatment unless they report any major side effects or decreased efficacy from carbidopa/levodopa and botulinum toxin injections.

In cases where pharmacological treatments are not effective, deep brain stimulation (DBS) surgery is proposed. DBS to the internal globus pallidus has been previously used as a treatment for primary generalized dystonia and has presented a degree of improvement in the range of 21%-95% [1], making it a viable option if the current treatments lose efficacy on either patient.

## Conclusions

The clinical presentations of myoclonus-dystonia can be easily confused or misdiagnosed due to the combination of neurological and psychiatric symptoms. Early signs of movement disorders tend to be indicative of a genetic disorder and should be considered if the symptoms don’t match the differential diagnosis. Diagnostic algorithms have been created to aid in the process of finding the correct pathologies among the various hypokinetic movement disorders. This case is also a reminder of the importance of a family history; this family presented a case of MDS that had a maternal imprinting, which complicated diagnosis more than other movement disorders with an autosomal dominant inheritance pattern.
